# Dual-region deep learning model integrating prostate and peri-prostatic adipose tissue MRI features for bone metastasis prediction in prostate cancer

**DOI:** 10.3389/fonc.2026.1946971

**Published:** 2026-09-07

**Authors:** Peng Qin, Bohao Liu, Huabin Su, Ziqiao Wang, Zhengxu Lin, Weian Zhu, Chen Zou, Qian Cai, Jianjie Wu, Jiayu Zheng, Xiao Zhao, Xuan Wen, Yun Luo

**Affiliations:** Department of Urology, The Third Affiliated Hospital of Sun Yat-sen University, Guangzhou, China

**Keywords:** biomedical image processing, bone metastasis, deep learning, prediction model, prostate cancer

## Abstract

**Rationale and objectives:**

Bone metastasis (BM) is pivotal in prostate cancer (PCa) management. This study developed a multimodal model integrating MRI-derived deep features from the prostate gland (PG) and periprostatic adipose tissue (PPAT) with clinical variables for BM risk assessment.

**Methods:**

Retrospectively, 464 patients were recruited and randomly divided into training and internal test cohorts at a ratio of 7:3. Deep features were extracted from PG and PPAT regions on T2-weighted MRI using a pretrained ResNet-50 as a fixed feature extractor. Clinical, PG, and PPAT component models were developed using patient-level cross-validation. Their out-of-fold probabilities were integrated by a logistic-regression meta-learner to construct the Deep feature-based PG-PPAT-Clinical (DPPC) model. Model performance was evaluated using ROC-AUC, average precision, calibration analysis, decision-curve analysis, and SHAP analysis.

**Results:**

The DPPC model achieved ROC-AUCs of 0.922 (95% CI, 0.887–0.954) in the training cohort and 0.928 (95% CI, 0.864–0.978) in the internal test cohort. Its ROC-AUC was significantly higher than that of the Clinical model in the training cohort and the PPAT model in the internal test cohort, whereas the remaining pairwise differences were not statistically significant. At a probability threshold of 0.5, the internal-test sensitivity and specificity were 65.1% and 95.9%, respectively. The observed BM rates were 87.5% in the high-risk group and 13.9% in the low-risk group.

**Conclusion:**

By synergizing deep learning signatures from PG and PPAT with clinical factors, the DPPC model demonstrates promising performance for BM risk stratification, and external validation and further calibration assessment are required.

## 1 Introduction

Prostate cancer (PCa) is the second most common malignancy in men worldwide, posing a significant threat to male health (1). The skeleton is the most common site of distant metastasis in PCa (2), and the occurrence of bone metastasis (BM) will fundamentally alter the patient’s clinical staging, treatment strategy, and prognosis (3, 4). Because the diagnostic yield of bone imaging varies across clinical risk groups and prostate-specific antigen (PSA) lacks sufficient individual specificity, improved noninvasive BM risk assessment could support initial-staging decisions (5–7).

In recent years, the critical role of the tumor microenvironment (TME) in cancer progression has garnered increasing attention (8). Distant metastasis is regarded as a complex biological process driven by the dynamic interplay between the “seed” (tumor cells) and the “soil” (microenvironment) (9). As a unique adipose reservoir enveloping the prostate gland (PG), periprostatic adipose tissue (PPAT) functions not merely as a structural support but as a metabolically active endocrine organ (10). It directly participates in remodeling the TME through paracrine pathways, thereby promoting tumor invasion and metastasis (11, 12). The accumulation of PPAT showed promising predictive value regarding the aggressiveness, metastasis, and prognosis of PCa (13, 14).

Current predictive models of BM are constrained by limitations in both imaging regions and methodologies. Previous studies have focused mainly on tumor lesions, while lacking information about the TME (15, 16). Although previous research focused on PPAT, it did not fully integrate the characteristics of the tumor proper (17). Solely analyzing any single region may overlook critical information. Furthermore, prior investigations primarily relied on traditional radiomics, yet these hand-crafted features are often limited in their ability to capture the deep, high-dimensional heterogeneity within the tissue.

In this study, the whole PG and PPAT were used as regions of interest. We integrated deep features from these regions with clinical variables to construct the Deep feature-based PG-PPAT-Clinical (DPPC) model. The aim was to develop and internally evaluate the model for BM risk assessment at initial staging and to examine the relative contributions of its three component-model probabilities.

## 2 Methods

### 2.1 Patient selection and data collection

**Figure 1:**
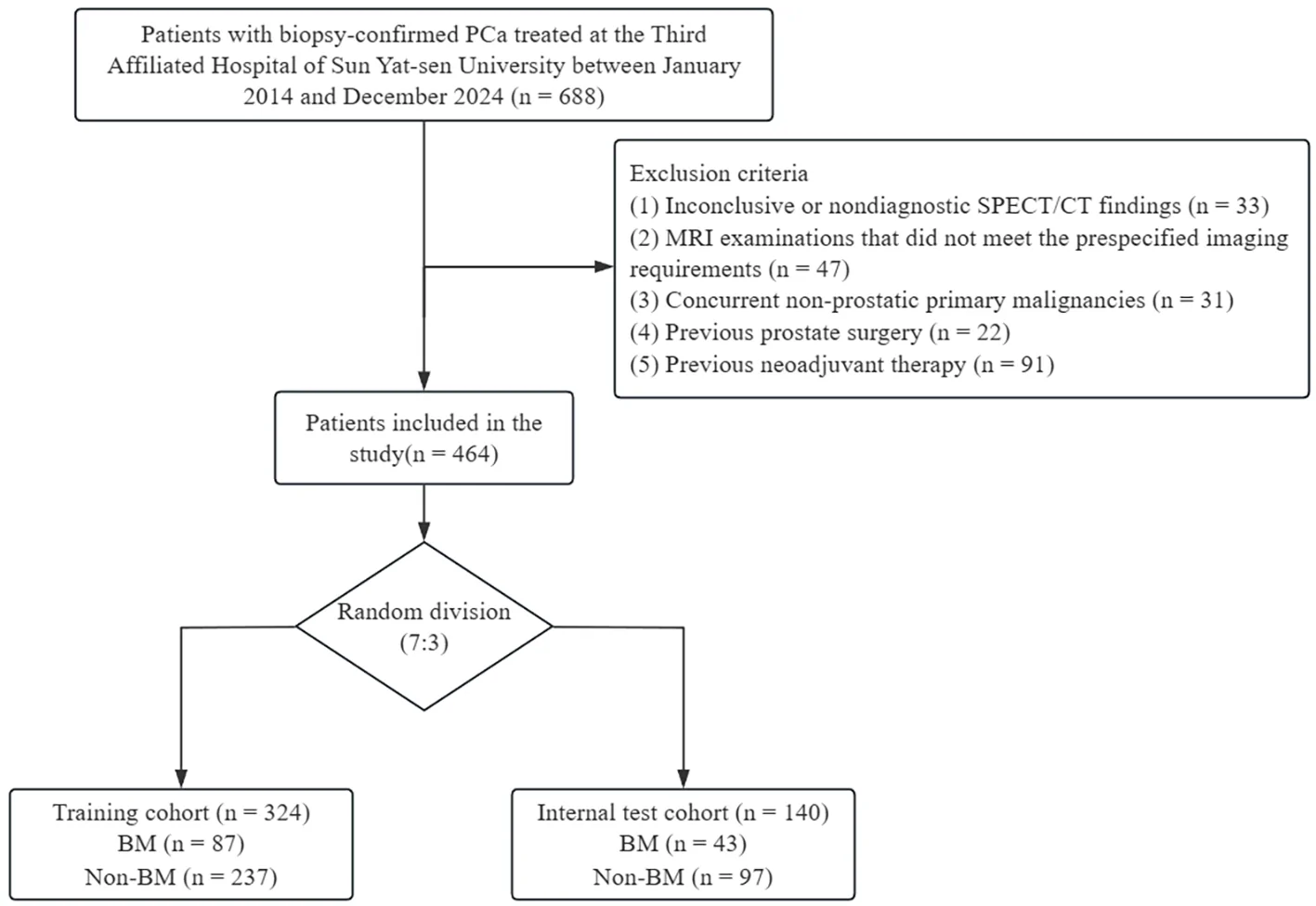
Flowchart of patient inclusion and grouping. PCa, prostate cancer; BM, bone metastasis, non-BM, without bone metastasis.

Ethical clearance for this retrospective investigation was granted by the Institutional Review Board of the authors’ hospital, which waived the requirement for informed consent (registration number: II2024-147-02). The study cohort comprised 688 patients with biopsy-confirmed PCa recruited from the Department of Urology between January 2014 and December 2024. The data collection and retrospective analysis were conducted in July 2025. Following diagnosis, all subjects underwent Technetium-99m methylene diphosphonate SPECT/CT imaging. We aggregated a comprehensive set of clinicopathologic variables, including age, body mass index (BMI), serum calcium and phosphorus levels, clinical T/N staging, comorbidities, and pelvic MRI findings. The ground truth for BM was established by the Nuclear Medicine Department based on tracer uptake patterns and morphological features on SPECT/CT. Exclusion criteria included (1): nondiagnostic or artifact-degraded SPECT/CT images (2); MRI examinations that did not provide axial T2-weighted images with a 3-mm slice thickness and complete coverage of the PG and PPAT from the bladder neck to the prostate apex (3); non-prostatic primary malignancies (4); prostate surgical history; or (5) history of neoadjuvant therapy. **Figure 1** illustrates the patient selection workflow.

A total of 464 eligible patients were included. Patients were randomly assigned to the training cohort (n = 324) or internal test cohort (n = 140) in a 7:3 ratio using random seed 42. The split was not stratified. Data partitioning and all cross-validation procedures were conducted at the patient level, with all images, slices, ROIs, and derived features from the same patient retained in the same partition.

The study cohort partially overlapped with two previous PPAT studies from our institution (17, 18), as detailed in **Supplementary Table 7**.

### 2.2 Reference standard and study chronology

BM status was determined from technetium-99m methylene diphosphonate SPECT/CT by two nuclear medicine physicians with 5 and 15 years of experience, respectively. They independently reviewed the examinations while blinded to MRI findings, clinical information, model outputs, and each other’s assessments. Discordant or equivocal findings were resolved by consensus; unresolved cases were adjudicated by a third senior nuclear medicine physician. Additional CT, MRI, follow-up imaging, pathological findings, or treatment response was considered when available. Pelvic MRI was generally performed approximately 2 weeks before prostate biopsy, and SPECT/CT was generally performed approximately 2 weeks after biopsy. All examinations used in this study were obtained during initial staging and before treatment.

### 2.3 MRI acquisition and ROI segmentation

Pelvic MRI scans were obtained using 1.5T or 3.0T platforms within three months prior to prostate biopsy and were generally obtained approximately 2 weeks before biopsy. The main acquisition parameters are provided in **Supplementary Table 9**. Two distinct ROIs were defined: PG region and PPAT region. Manual segmentation was performed on 3-mm axial T2-weighted slices extending from the bladder neck to the prostate apex, utilizing 3D Slicer software (version 5.2.1). The segmentation was initially performed by a radiologist with 5 years of experience and subsequently reviewed and refined by a senior radiologist with 15 years of experience. For the PG region, the ROI was delineated to encompass the entire prostatic parenchyma along the capsule from the base to the apex, strictly excluding the seminal vesicles. For the PPAT region, in accordant with previously described criteria (13), the delineated volume included the anterior venous plexus and prostate-draining vessels, while strictly excluding mesorectal fat. The anatomical boundaries were defined as follows (1): anteriorly by the pubic symphysis (2); laterally by the fascial boundary adjacent to the levator ani muscles; and (3) posteriorly by Denonvilliers’ fascia.

### 2.4 Volumetric quantification and reliability

Prostate and PPAT volumes were quantified by multiplying the cumulative cross-sectional ROI areas by the slice thickness. A normalized PPAT volume was subsequently derived as the ratio of PPAT volume to prostate volume (17). For the reproducibility assessment, two radiologists independently completed PG and PPAT segmentation in a prespecified subset of 30 patients from the training cohort. Interobserver reliability was quantified using a two-way random-effects, absolute-agreement, single-measure intraclass correlation coefficient [ICC (1, 2)] with 95% confidence intervals using the `irr::icc()` function in R version 4.3.1. The reliability assessment was performed once before model development.

### 2.5 Clinical model construction

The Clinical model included tPSA, serum alkaline phosphatase (ALP), clinical T stage (cT), clinical N stage (cN), and biopsy-based ISUP grade group. To reduce redundancy among PSA-related variables, only tPSA was retained and was categorized as <10, 10–20, or >20 ng/mL. ALP was log2-transformed, whereas cT and ISUP grade group were treated as categorical variables, with cT1 and ISUP grade group 1 as the respective reference categories; cN was treated as a binary variable. The functional forms of continuous predictors were assessed by comparing linear terms with natural cubic splines using likelihood-ratio tests. The Clinical model was constructed using multivariable logistic regression. During five-fold cross-validation, the model was fitted within each training fold and applied to the corresponding held-out patients to generate out-of-fold probabilities. After cross-validation, the model was refitted using the entire training cohort and applied to the internal test cohort.

### 2.6 Image preprocessing and deep learning feature extraction

To standardize spatial dimensions, all images were resized to a uniform resolution of 224 × 224 pixels using linear interpolation. Channel-wise pixel intensities were normalized to a mean and standard deviation of 0.5 to comport with the input specifications of the deep learning framework. We utilized a ResNet-50 architecture, pretrained on the ImageNet dataset (http://www.image-net.org), as the backbone for feature extraction within the PyTorch environment. To ensure a stable and objective feature extraction process while maintaining computational efficiency, the pretrained weights were kept frozen, functioning as a fixed feature extractor. This strategy avoids the extensive computational overhead and potential overfitting risks associated with iterative fine-tuning on a relatively small medical dataset. A customized module was employed to extract feature vectors from the global average pooling layer, ensuring the capture of deep semantic representations for each image. Detailed hyperparameters, preprocessing steps, and aggregation protocols are provided in the Supplementary Method S1. Slice-level feature vectors were averaged to obtain one patient-level representation for each region.

### 2.7 Feature selection and model construction

Feature reproducibility was assessed once in a prespecified selected subset of 30 patients from the training cohort. Features with an ICC greater than 0.6 were retained as the fixed candidate feature set for subsequent model development.

The training cohort was divided into five patient-level outer folds. Within each outer-training fold, Z-score normalization, univariable screening with multiple-testing correction, Pearson correlation filtering, and LASSO selection with 10-fold inner cross-validation were performed separately for the PG and PPAT features. The parameters and selected features obtained from each outer-training fold were subsequently applied to its corresponding held-out fold.

**Figure 2:**
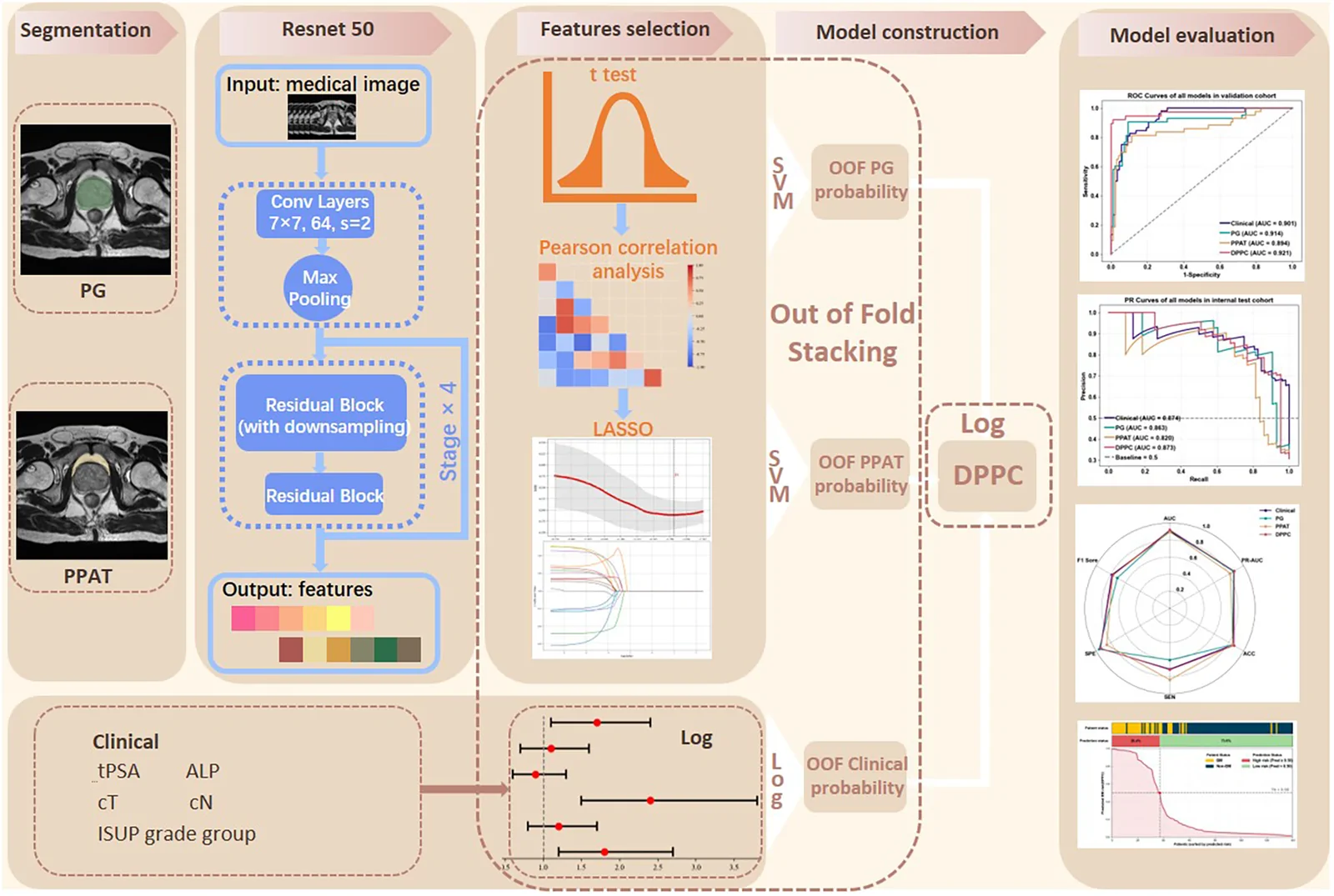
Workflow of the study. Deep features extracted from the PG and PPAT using a pretrained ResNet-50 were combined with clinical predictors. Patient-level out-of-fold probabilities from the Clinical, PG, and PPAT models were integrated using logistic-regression stacking. Training-cohort DPPC performance was estimated by meta-level cross-fitting, and the fitted pipeline was evaluated in the internal test cohort. PG, prostate gland; PPAT, periprostatic adipose tissue; DPPC, Deep feature-based PG-PPAT-Clinical model; LASSO, least absolute shrinkage and selection operator; SVM, support-vector machine; OOF, out-of-fold.

PG and PPAT signatures were used to construct separate SVM classifiers. Within each outer-training fold, SVM hyperparameters were optimized by inner cross-validation, class_weight = “balanced” was used to account for class imbalance, and posterior probabilities were calibrated using Platt scaling. The resulting fold-specific pipelines generated calibrated PG and PPAT probabilities for the corresponding held-out patients. The complete patient-level out-of-fold stacking and meta-level cross-fitting procedure is summarized in **Figure 2** and detailed in Supplementary Algorithm S1 in the **Supplementary Material**.

### 2.8 Probability stacking

In each outer iteration, the Clinical, PG, and PPAT pipelines generated probabilities for the corresponding held-out patients. After completion of the five outer folds, the three out-of-fold probabilities available for each patient in the training cohort were used as predictors in a logistic-regression meta-learner to construct the DPPC model. The three base-model pipelines were subsequently refitted using the entire training cohort and applied to the internal test cohort. The resulting Clinical, PG, and PPAT probabilities were entered into the fitted logistic-regression meta-learner to obtain the final DPPC probability. Training-cohort performance of the DPPC model was estimated using a second patient-level five-fold cross-fitting procedure at the meta-learner level, as detailed in Algorithm S1 in the **Supplementary Material**.

### 2.9 Model evaluation and interpretability

Model discrimination was evaluated using receiver-operating-characteristic (ROC) curves, ROC-AUC, precision-recall curves, and average precision (AP). ROC-AUC, AP, accuracy, sensitivity, specificity, F1 score, positive predictive value, and negative predictive value were reported with 95% confidence intervals estimated using 10, 000 patient-level outcome-stratified nonparametric bootstrap replicates. For descriptive classification analyses, a fixed probability threshold of 0.5 was applied uniformly to all models. Pairwise ROC-AUC differences between the DPPC model and each component model were evaluated using two-sided DeLong tests for correlated ROC curves. IDI values, percentile 95% confidence intervals, and two-sided empirical P values were estimated using 10, 000 patient-level outcome-stratified nonparametric bootstrap replicates. Detailed statistical procedures are provided in Supplementary Method S2. Calibration was assessed using calibration curves based on four equal-frequency probability groups, Brier score, expected calibration error (ECE), calibration intercept and slope, and the Hosmer-Lemeshow test using 10 equal-frequency groups. Bootstrap 95% confidence intervals were calculated for the calibration curves, Brier score, ECE, calibration intercept, and calibration slope. Decision-curve analysis was performed across the evaluated threshold-probability range, with bootstrap 95% confidence bands for net benefit. SHAP analysis was used to quantify the contributions of the Clinical, PG, and PPAT component-model probabilities to the output of the logistic-regression meta-learner. The overall workflow encompassing feature extraction, model construction, and model evaluation is illustrated in **Figure 2**.

### 2.10 Statistical analysis

Continuous variables were reported as means ± standard deviations or medians with interquartile ranges, contingent upon data distribution. Categorical data were summarized as frequencies and percentages. The overall training and internal test cohorts were compared using Welch’s t test, the Mann-Whitney U test, Pearson’s chi-square test, or Fisher’s exact test, as appropriate. Comparisons by BM status within each cohort were descriptive and were not used for predictor selection. Statistical tests were two-sided, with P <0.05 considered statistically significant. Analyses were performed using R version 4.3.1 and Python version 3.12.13; the DeLong and bootstrap analyses used pandas version 3.0.1 and NumPy version 2.3.5.

## 3 Results

### 3.1 Patient characteristics and recruitment

The study enrolled 464 eligible patients (**Figure 1**), with 324 assigned to training cohort (BM, n = 87) and 140 to internal test cohort (BM, n = 43). The overall cohorts were similar for most variables, although the distributions of cT stage (P = 0.004) and PI-RADS category (P <0.001) differed (**Table 1**). Within both cohorts, BM was associated with higher tPSA, fPSA, PSAD, ALP, and normalized PPAT volume and with different distributions of tPSA category, cT stage, cN stage, ISUP grade group, and PI-RADS category (**Supplementary Table 1**).

**Table 1:** Clinical characteristics of the training and internal test cohorts.

Variable	Training cohort (n = 324)	Internal test cohort (n = 140)	P value
Age, years	70.5 ± 8.7	70.8 ± 8.7	0.712
BMI, kg/m²	23.32 (21.23–25.39)	23.10 (20.82–25.10)	0.578
tPSA, ng/mL	20.36 (10.25–65.12)	28.78 (10.79–79.72)	0.217
fPSA, ng/mL	2.90 (1.36–8.02)	3.05 (1.57–12.46)	0.320
f/tPSA	0.14 (0.10–0.21)	0.14 (0.10–0.22)	0.777
PSAD, ng/mL/cm³	0.53 (0.22–1.34)	0.70 (0.23–1.87)	0.129
ALP, U/L	68.00 (56.00–85.00)	69.00 (56.75–89.75)	0.681
Prostate volume, cm³	40.71 (28.82–57.98)	39.28 (29.16–53.22)	0.554
Normalized PPAT volume	0.84 (0.63–1.17)	0.85 (0.65–1.07)	0.271
Serum calcium, mmol/L	2.32 (2.22–2.41)	2.33 (2.25–2.41)	0.739
Serum phosphorus, mmol/L	1.09 (0.98–1.20)	1.07 (0.96–1.21)	0.467
**tPSA category**, **ng/mL**			**0.359**
<10	75 (23.1%)	31 (22.1%)	
10–20	85 (26.2%)	29 (20.7%)	
>20	164 (50.6%)	80 (57.1%)	
**Clinical T stage**			**0.004**
1	41 (12.7%)	20 (14.3%)	
2	161 (49.7%)	49 (35.0%)	
3	63 (19.4%)	26 (18.6%)	
4	59 (18.2%)	45 (32.1%)	
**Clinical N stage**			**0.686**
0	210 (64.8%)	88 (62.9%)	
1	114 (35.2%)	52 (37.1%)	
**ISUP grade group**			**0.089**
1	60 (18.5%)	23 (16.4%)	
2	36 (11.1%)	12 (8.6%)	
3	53 (16.4%)	12 (8.6%)	
4	78 (24.1%)	45 (32.1%)	
5	97 (29.9%)	48 (34.3%)	
**PI-RADS category**			**<0.001**
1	7 (2.2%)	0 (0.0%)	
2	21 (6.5%)	17 (12.1%)	
3	65 (20.1%)	14 (10.0%)	
4	107 (33.0%)	35 (25.0%)	
5	124 (38.3%)	74 (52.9%)	
**Diabetes**			**0.465**
No	289 (89.2%)	128 (91.4%)	
Yes	35 (10.8%)	12 (8.6%)	
**Hypertension**			**0.980**
No	238 (73.5%)	103 (73.6%)	
Yes	86 (26.5%)	37 (26.4%)	
**Coronary heart disease**			**0.926**
No	286 (88.3%)	124 (88.6%)	
Yes	38 (11.7%)	16 (11.4%)	

Age is reported as mean ± standard deviation; other continuous variables are reported as median (interquartile range). Categorical variables are reported as n (column %). There were no missing values for the variables shown. P values are descriptive and were not used for predictor selection. BM, bone metastasis; BMI, body mass index; tPSA, total prostate-specific antigen; fPSA, free prostate-specific antigen; PSAD, prostate-specific antigen density; ALP, alkaline phosphatase; PPAT, periprostatic adipose tissue; ISUP, International Society of Urological Pathology; PI-RADS, Prostate Imaging Reporting and Data System. Bold type indicates categorical variable headings and the corresponding overall P values.

### 3.2 Clinical model results

The likelihood-ratio test indicated a nonlinear association between tPSA and the logit of BM (P = 0.011), supporting the use of clinically defined tPSA categories. No evidence of nonlinearity was observed for log2-transformed ALP (P = 0.753). The maximum VIF and condition index were 2.93 and 27.88, respectively, indicating no severe multicollinearity in the Clinical model. Detailed correlation matrices, VIFs, condition indices, functional-form assessments, and the complete Clinical-model equation are provided in **Supplementary Tables S2**–**S6**. The complete model coefficients are presented in **Table 2**.

**Table 2:** Multivariable logistic-regression coefficients of the prespecified clinical model.

Predictor	β coefficient	Standard error	Odds ratio	95% CI	P value
Intercept	-11.175	2.318	—	—	<0.001
tPSA 10–20 ng/mL	-0.427	0.513	0.653	0.239–1.785	0.406
tPSA >20 ng/mL	0.197	0.446	1.218	0.508–2.916	0.659
log2(ALP)	1.087	0.312	2.964	1.607–5.469	<0.001
cT2	2.018	1.069	7.527	0.926–61.180	0.059
cT3	1.968	1.109	7.159	0.815–62.884	0.076
cT4	3.303	1.119	27.192	3.035–243.611	0.003
cN positive	0.794	0.348	2.213	1.120–4.373	0.022
ISUP grade group 2	0.713	0.754	2.041	0.465–8.954	0.344
ISUP grade group 3	0.751	0.670	2.120	0.570–7.890	0.262
ISUP grade group 4	1.000	0.636	2.719	0.781–9.460	0.116
ISUP grade group 5	1.102	0.630	3.010	0.875–10.357	0.080

Reference categories were tPSA <10 ng/mL, cT1, cN negative, and ISUP grade group 1. ALP was entered after log2 transformation. All predictors were entered simultaneously without univariable P-value screening. CI, confidence interval; tPSA, total prostate-specific antigen; ALP, alkaline phosphatase; cT, clinical T stage; cN, clinical N stage; ISUP, International Society of Urological Pathology.

### 3.3 Feature extraction and selection

The rigorous feature selection workflow is visually detailed in **Figure 2**. Initially, a total of 2048 deep learning features were extracted from the PG and PPAT regions, respectively. The ICC analysis performed in 30 patients from the training cohort retained 860 PG and 276 PPAT features. Feature selection was conducted separately within each outer-training fold during out-of-fold model development. When the pipelines were refitted using the entire training cohort, univariable screening and correlation filtering retained 198 PG and 160 PPAT features, and LASSO regression ultimately selected 16 PG and 15 PPAT features for the final models (**Supplementary Figures S1** and **S2**). The ICCs for PG and PPAT volume were 0.856 and 0.887, respectively.

### 3.4 Model construction and performance

**Figure 3:**
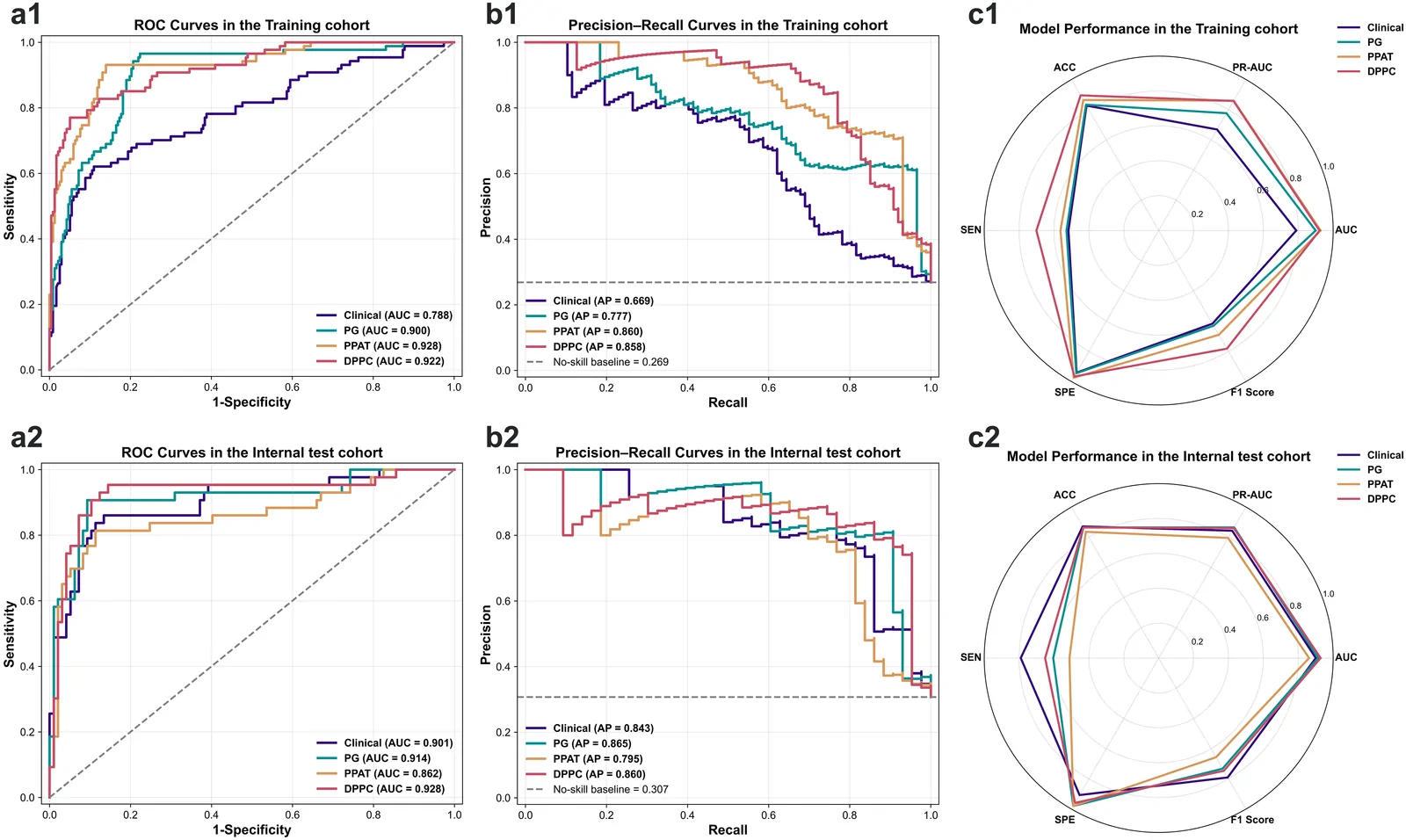
Performance evaluation of different models in the training and internal test cohorts. **(a)** Receiver operating characteristic curves; **(b)** precision–recall curves; and **(c)** radar charts summarizing model performance metrics. PG, prostate gland; PPAT, periprostatic adipose tissue; DPPC, Deep feature-based PG-PPAT-Clinical model; AUC, area under the curve; ACC, accuracy; SEN, sensitivity; SPE, specificity.

The performance of the Clinical, PG, PPAT, and DPPC models is summarized in **Table 3** and **Figure 3**. DPPC achieved ROC-AUCs of 0.922 (95% CI, 0.887–0.954) in the training cohort and 0.928 (95% CI, 0.864–0.978) in the internal test cohort. The corresponding AP values were 0.858 (95% CI, 0.795–0.917) and 0.860 (95% CI, 0.754–0.954), respectively.

**Table 3:** Performance of different models in the training and internal test cohorts.

Performance measure	Clinical	PG	PPAT	DPPC
Training cohort				
ROC-AUC (95% CI)	0.788 (0.726–0.849)	0.900 (0.859–0.935)	0.928 (0.893–0.959)	0.922 (0.887–0.954)
AP (95% CI)	0.669 (0.583–0.759)	0.777 (0.704–0.848)	0.860 (0.802–0.914)	0.858 (0.795–0.917)
Accuracy (95% CI)	0.827 (0.790–0.861)	0.833 (0.796–0.867)	0.864 (0.833–0.895)	0.895 (0.864–0.926)
Sensitivity (95% CI)	0.517 (0.414–0.621)	0.529 (0.425–0.632)	0.563 (0.460–0.667)	0.701 (0.609–0.793)
Specificity (95% CI)	0.941 (0.911–0.970)	0.945 (0.916–0.975)	0.975 (0.954–0.992)	0.966 (0.941–0.987)
F1 score (95% CI)	0.616 (0.519–0.705)	0.630 (0.534–0.716)	0.690 (0.598–0.772)	0.782 (0.709–0.850)
PPV (95% CI)	0.763 (0.667–0.863)	0.780 (0.683–0.880)	0.891 (0.808–0.963)	0.884 (0.812–0.953)
NPV (95% CI)	0.842 (0.813–0.872)	0.845 (0.816–0.876)	0.859 (0.830–0.889)	0.898 (0.869–0.928)
TP/TN/FP/FN	45/223/14/42	46/224/13/41	49/231/6/38	61/229/8/26
Internal test cohort				
ROC-AUC (95% CI)	0.901 (0.835–0.955)	0.914 (0.849–0.966)	0.862 (0.780–0.934)	0.928 (0.864–0.978)
AP (95% CI)	0.843 (0.753–0.923)	0.865 (0.779–0.943)	0.795 (0.683–0.906)	0.860 (0.754–0.954)
Accuracy (95% CI)	0.871 (0.814–0.921)	0.864 (0.814–0.914)	0.836 (0.786–0.886)	0.864 (0.814–0.914)
Sensitivity (95% CI)	0.791 (0.674–0.907)	0.605 (0.465–0.744)	0.512 (0.372–0.651)	0.651 (0.512–0.791)
Specificity (95% CI)	0.907 (0.845–0.959)	0.979 (0.948–1.000)	0.979 (0.948–1.000)	0.959 (0.918–0.990)
F1 score (95% CI)	0.791 (0.696–0.874)	0.732 (0.606–0.838)	0.657 (0.508–0.778)	0.747 (0.629–0.847)
PPV (95% CI)	0.791 (0.689–0.900)	0.929 (0.828–1.000)	0.917 (0.806–1.000)	0.875 (0.765–0.970)
NPV (95% CI)	0.907 (0.858–0.957)	0.848 (0.803–0.897)	0.819 (0.775–0.866)	0.861 (0.814–0.913)
TP/TN/FP/FN	34/88/9/9	26/95/2/17	22/95/2/21	28/93/4/15

All confidence intervals were estimated using 10, 000 patient-level outcome-stratified nonparametric bootstrap replicates. Classification metrics were calculated at the fixed probability threshold of 0.5. ROC-AUC, area under the receiver operating characteristic curve; AP, average precision; PPV, positive predictive value; NPV, negative predictive value; PG, prostate gland; PPAT, periprostatic adipose tissue; DPPC, deep feature-based PG-PPAT-Clinical model.

In the training cohort, the DPPC ROC-AUC was higher than that of the Clinical model (0.922 vs 0.788; P <0.001) but did not differ significantly from the PG (P = 0.213) or PPAT (P = 0.626) ROC-AUC. In the internal test cohort, DPPC had the highest point-estimate ROC-AUC; the difference was significant relative to PPAT (0.928 vs 0.862; P = 0.018) but not relative to the Clinical (P = 0.287) or PG (P = 0.429) model (**Table 4**).

**Table 4:** Performance comparison between DPPC and other models.

Cohort	Comparison	ROC-AUC	DeLong Z	DeLong P	IDI (95% CI)	IDI P
Training cohort	DPPC vs Clinical	0.922 vs 0.788	5.448	<0.001	0.249 (0.184–0.315)	<0.001
Training cohort	DPPC vs PG	0.922 vs 0.900	1.246	0.213	0.227 (0.176–0.278)	<0.001
Training cohort	DPPC vs PPAT	0.922 vs 0.928	-0.488	0.626	0.149 (0.116–0.182)	<0.001
Internal test cohort	DPPC vs Clinical	0.928 vs 0.901	1.064	0.287	0.070 (-0.009–0.146)	0.081
Internal test cohort	DPPC vs PG	0.928 vs 0.914	0.791	0.429	0.161 (0.102–0.223)	<0.001
Internal test cohort	DPPC vs PPAT	0.928 vs 0.862	2.370	0.018	0.182 (0.139–0.225)	<0.001

ROC-AUC comparisons used two-sided paired DeLong tests. IDI confidence intervals and two-sided empirical P values used 10, 000 patient-level outcome-stratified nonparametric bootstrap replicates. ROC-AUC, area under the receiver operating characteristic curve; IDI, integrated discrimination improvement; CI, confidence interval; PG, prostate gland; PPAT, periprostatic adipose tissue; DPPC, Deep feature-based PG-PPAT-Clinical model.

IDI values for DPPC relative to the Clinical, PG, and PPAT models in the training cohort were 0.249 (95% CI, 0.184–0.315), 0.227 (95% CI, 0.176–0.278), and 0.149 (95% CI, 0.116–0.182), respectively (all P <0.001). In the internal test cohort, the corresponding IDI values were 0.070 (95% CI, −0.009–0.146; P = 0.081), 0.161 (95% CI, 0.102–0.223; P <0.001), and 0.182 (95% CI, 0.139–0.225; P <0.001). Thus, these results indicated improved discrimination slopes for the DPPC model, although statistically significant ROC-AUC improvements were not observed in all pairwise comparisons (**Table 4**).

### 3.5 Model calibration and clinical usefulness

**Figure 4:**
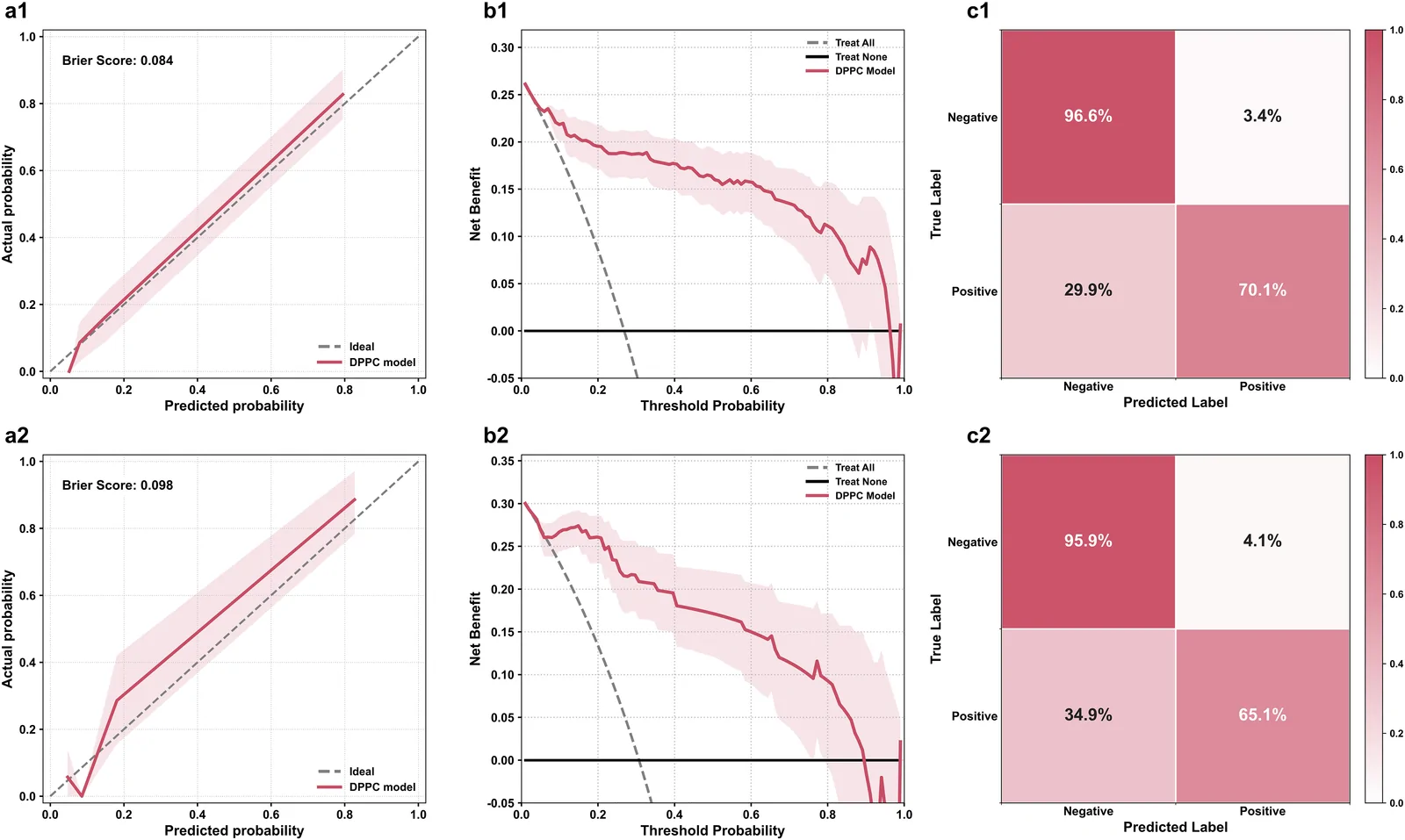
Calibration, decision-curve, and classification analyses of DPPC. **(a)** Calibration curves constructed using four equal-frequency groups, with bootstrap 95% confidence bands; **(b)** decision curves with bootstrap 95% confidence bands; and **(c)** row-normalized confusion matrices at the fixed probability threshold of 0.5.

Calibration curves based on four equal-frequency groups are shown in **Figure 4a**. The Brier scores were 0.084 (95% CI, 0.064–0.105) in the training cohort and 0.098 (95% CI, 0.067–0.133) in the internal test cohort. The corresponding ECE values were 0.026 (95% CI, 0.020–0.058) and 0.065 (95% CI, 0.039–0.110). Calibration intercepts were 0.118 (95% CI, −0.290–0.777) and 0.320 (95% CI, −0.287–2.538), and calibration slopes were 1.093 (95% CI, 0.868–1.474) and 1.075 (95% CI, 0.717–2.353), respectively. Hosmer-Lemeshow P values were 0.058 and 0.009. Although the Brier scores were relatively low and the calibration slopes were close to 1, the internal-test calibration curve showed localized deviations, and the confidence intervals for the calibration parameters were wide. Decision curves with bootstrap confidence bands are shown in **Figure 4b**. The estimated net benefit of DPPC was positive across much of the evaluated threshold range.

**Figure 5:**
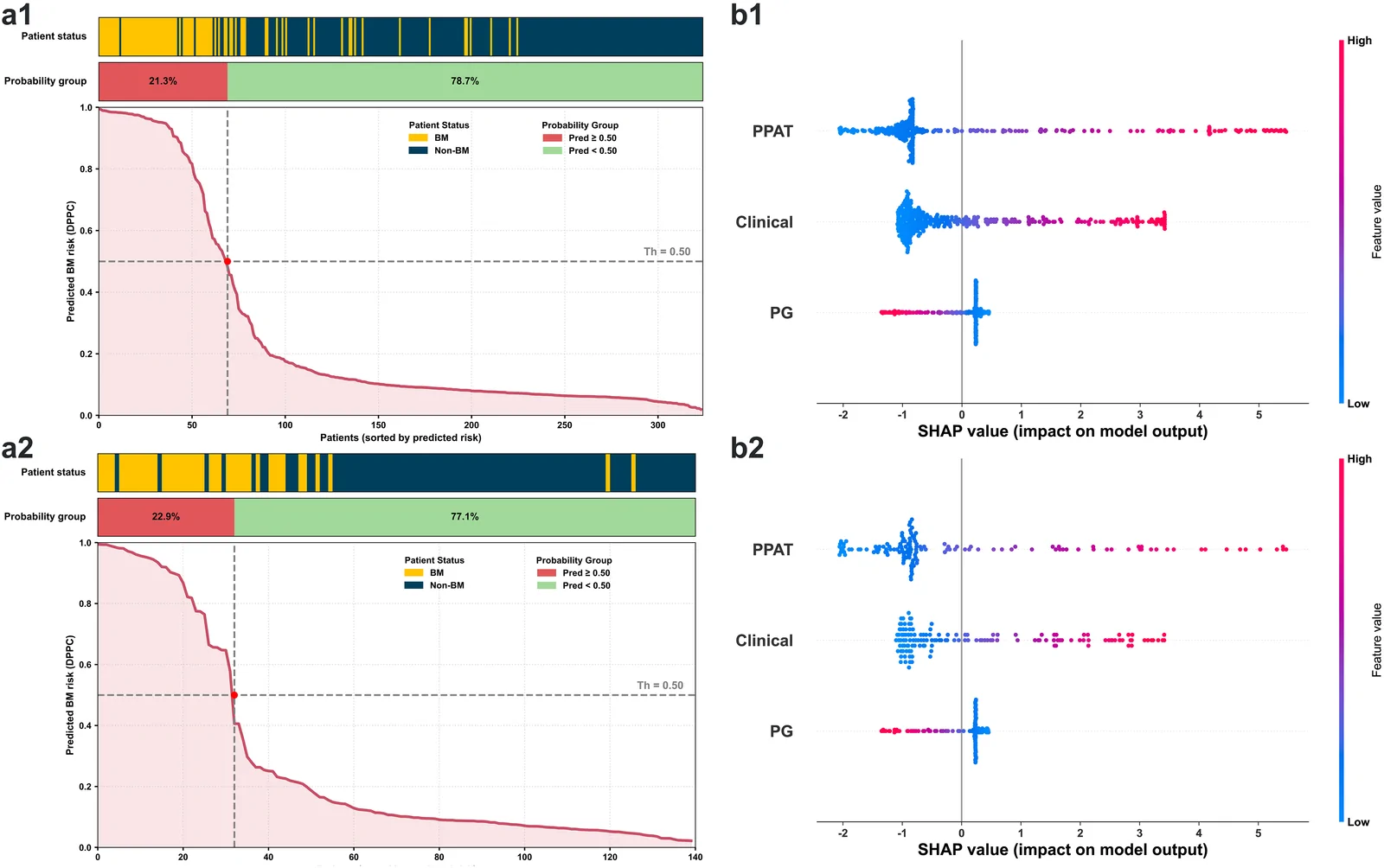
Risk stratification and SHAP beeswarm plot of DPPC model in training and internal test cohorts. **(a)** Patients ordered by predicted BM probability, with probability groups defined descriptively by the fixed threshold of 0.5; **(b)** SHAP beeswarm plots showing the contributions of the Clinical-, PG-, and PPAT-model probabilities to the logistic-regression meta-learner output. PG, prostate gland; PPAT, periprostatic adipose tissue; DPPC, Deep feature-based PG-PPAT-Clinical model; BM, bone metastasis, non-BM, without bone metastasis.

A probability threshold of 0.5 was used for the classification and risk-stratification analyses. In the training cohort, the model correctly identified 70.1% of positive cases and 96.6% of negative cases. In the internal test cohort, the model achieved true positive and true negative rates of 65.1% and 95.9% (**Figure 4c**). Furthermore, the DPPC model facilitated effective risk stratification for BM (**Figure 5a**). In the training cohort, BM was present in 61 of 69 patients classified as high risk (88.4%) and 26 of 255 patients classified as low risk (10.2%). In the internal test cohort, BM was present in 28 of 32 high-risk patients (87.5%) and 15 of 108 low-risk patients (13.9%). The BM rates differed significantly between the high- and low-risk groups in both cohorts (both P < 0.001). An alternative threshold of 0.109675, selected from the training-cohort out-of-fold predictions to retain a sensitivity of at least 90%, was applied unchanged to the internal test cohort; the corresponding results are provided in **Supplementary Table 8**.

### 3.6 Model interpretability

SHAP analysis showed that the PPAT-model probability had the largest mean absolute contribution to the DPPC log-odds output in both cohorts, followed by the Clinical- and PG-model probabilities (**Figure 5b**). The color distribution patterns indicate that higher scores (red dots) for PPAT and Clinical are positively correlated with positive SHAP values, suggesting that elevated levels of these markers significantly increase the predicted risk of BM. Higher PPAT- and Clinical-model probabilities generally contributed positively to the DPPC output.

## 4 Discussion

In this study, we developed and internally evaluated the DPPC model, which integrates deep features from the PG and PPAT with clinical variables for BM risk assessment in patients with PCa. The DPPC model achieved ROC-AUCs of 0.922 in the training cohort and 0.928 in the internal test cohort. In the training cohort, its ROC-AUC was significantly higher than that of the Clinical model but did not differ significantly from those of the PG and PPAT models. In the internal test cohort, the DPPC model had the highest point-estimate ROC-AUC, although a statistically significant difference was observed only in comparison with the PPAT model. The point estimates suggest that integrating the three component-model probabilities may provide complementary predictive information. By integrating information from the PG, PPAT, and clinical variables, the DPPC model provides an individualized BM risk estimate for initial staging.

BM is a major contributor to morbidity and mortality in patients with PCa (19) and represents pivotal clinical implications in treatment decision-making (4). In the internal test cohort, the 0.5 threshold identified a subgroup with a high probability of BM, with a specificity of 95.9% and an observed BM rate of 87.5% among patients classified as high risk. This operating point is therefore more suitable for identifying patients with a high metastatic risk. More broadly, the continuous probability generated by DPPC integrates complementary clinical, PG, and PPAT information and may support individualized risk assessment during initial staging. Prospective multicenter validation and further evaluation of threshold-specific clinical utility are required before clinical implementation.

A distinct methodological advantage of our study lies in the strategic selection of the ROI. Unlike prior MRI-based radiomics models that primarily focused on delineating specific intraprostatic tumor lesions (20, 21), we utilized the whole PG as the ROI. This approach addresses twofold limitations inherent in lesion-based methods regarding clinical translation. First, at the operational level, lesion-based models rely on the precise identification of tumor boundaries. Given that PCa is often multifocal and some lesions appear isointense on MRI, lesion identification and segmentation are prone to high subjectivity and interobserver variability (22, 23). In contrast, the anatomical boundaries of the whole PG are well-defined, allowing urologists to achieve accurate, reproducible segmentation after only brief training (24). Furthermore, analyzing the entire PG ensures the capture of comprehensive information from occult or multifocal lesions, this holistic approach has been validated to enhance diagnostic accuracy in various malignancies (24–26). Second, focusing solely on the tumor lesion often leads to “tunnel version, “ neglecting the interaction between the tumor and its surrounding microenvironment. Specifically, PPAT, serving as the “soil” adjacent to the prostate, plays a non-negligible role in tumor progression.

From a biological perspective, the performance of the DPPC model may partly reflect the complementary information provided by the PG, PPAT, and clinical factors within the conceptual framework of the “seed-and-soil” interaction. The PG is intimately associated with PPAT, with nearly one-third of its anterior surface in direct contact. PPAT not only influences PCa metabolism but also functions as a metabolically active endocrine organ that profoundly affects disease progression via multiple mechanisms (13, 27, 28). Biological studies have demonstrated that PPAT secretes adipokines such as chemokine (C-C motif) ligand 7, interleukin-6, and leptin. These factors not only promote tumor angiogenesis but also induce epithelial-mesenchymal transition and remodel the extracellular matrix, thereby creating a “pro-metastatic microenvironment” that facilitates tumor cell escape and distant BM (11, 29–31). Previous clinical studies also corroborate this: prostate invasion into PPAT is an adverse prognostic factor (32), and MRI radiomics features of PPAT are associated with clinically significant PCa and lymph node metastasis (33, 34). Previous work also confirmed that normalized PPAT volume is an independent predictor of BM (18), and a radiomics nomogram based on PPAT can predict BM in PCa (17). However, such interpretations remain hypotheses for future biological validation. In particular, the component-level SHAP analysis explains only the contributions of the PG-, PPAT-, and Clinical-model probabilities; it does not demonstrate that the extracted features represent microscopic phenotypes, metabolic alterations, or specific molecular pathways.

However, these hand-crafted traditional radiomics features in the prior models were often limited in capturing deep, non-linear, and high-dimensional tissue heterogeneity (35). In contrast, the deep learning technology employed in this study can automatically extract data-driven abstract features. Regarding the architecture selection, we employed a ResNet-50 backbone pretrained on ImageNet. A 2D slice-based extraction strategy was used to accommodate the anisotropic resolution of clinical MRI, and the slice-level features were subsequently averaged to obtain patient-level representations. Furthermore, considering the dataset size, using a pretrained model with frozen weights effectively balanced feature richness with the risk of overfitting. This capability allows it to capture subtle, invisible changes in the PPAT microenvironment more sensitively than traditional methods (36). Unlike predefined handcrafted features, these data-driven representations may encode complex imaging patterns within the PG and PPAT, although their biological significance remains to be established. The point-estimate results and positive IDI values suggest that probability-level fusion may add information in some comparisons, but the nonsignificant DeLong and IDI results in other comparisons require cautious interpretation.

Calibration was closer to the ideal line in the training cohort than in the internal test cohort. Although the Brier scores and calibration slopes indicated relatively low overall prediction error, the significant internal-test Hosmer-Lemeshow result, deviations in intermediate probability groups, and wide confidence intervals indicate remaining calibration uncertainty. Recalibration should be considered before clinical application in a new population.

For descriptive context, Lin et al. reported an AUC of 0.893 for an intratumoral and peritumoral radiomics model (37), Yang et al. reported an AUC of 0.862 for a prostate MRI-based deep learning model (38), and Zhang et al. reported an AUC of 0.890 for the MRI-based deep learning component of their multimodal framework (39). In the present study, the DPPC model yielded an AUC of 0.928 in the internal test cohort. However, differences in study populations, imaging protocols, reference standards, and validation designs preclude direct comparisons of model performance. Head-to-head evaluation of these models on the same independent external dataset is required to determine their relative performance.

Despite the promising results, this study has several limitations. First, it was a retrospective single-center study using a nonstratified random internal split rather than temporal or external validation. The internal test cohort contained only 43 BM events, and the cT and PI-RADS distributions differed between the two cohorts. Consequently, the results do not establish temporal, scanner-related, geographic, or population transportability. Multicenter external validation of a frozen pipeline, followed by site-specific calibration assessment, is required. Second, the long acquisition period and use of 1.5-T and 3.0-T platforms introduced potential scanner and protocol heterogeneity, which may have affected feature stability. Third, our reliance on manual ROI segmentation and single-sequence (T2WI) data may constrain efficiency and information depth. Future work should integrate fully automated segmentation and functional sequences to enhance clinical translation. Additionally, although PSMA PET-CT provides superior diagnostic accuracy, it was not routinely available throughout our study period; therefore, the reliance on SPECT/CT as the ground truth may have led to an underestimation of the micro-metastatic cases. Future investigations are warranted to benchmark the predictive performance of the DPPC model against PSMA PET-CT to further evaluate its clinical utility.

## 5 Conclusion

The DPPC model, which integrates deep learning signatures from both PG and PPAT regions with clinical variables, demonstrates good discrimination for BM in patients with newly diagnosed PCa. By providing individualized BM risk estimates, the model may support risk stratification during the initial staging of patients with newly diagnosed PCa, although further external validation is warranted.

## Data Availability

The original contributions presented in the study are included in the article/**Supplementary Material**. Further inquiries can be directed to the corresponding author.
