# Supplementary material for: Dual-region deep learning model integrating prostate and peri-prostatic adipose tissue MRI features for bone metastasis prediction in prostate cancer
Source: Front Oncol. 2026 Sep 7;16:1946971. doi: 10.3389/fonc.2026.1946971 (PMC13590367; doi:10.3389/fonc.2026.1946971)
Supplement: Supplementary file 1 [file SupplementaryFile1.docx]

Supplementary Material

# 1 Supplementary Methods

**Method S1.** MRI preprocessing and deep-feature extraction

Pelvic MRI was performed using 1.5-T or 3.0-T systems from GE Healthcare, Siemens Healthineers, and United Imaging Healthcare. Axial T2-weighted images were used for PG and PPAT segmentation and subsequent deep-feature extraction. The main acquisition parameters are summarized in Table S9.

Axial T2-weighted ROI images were resized to 224 × 224 pixels using linear interpolation. Channel-wise intensities were normalized using a mean and standard deviation of 0.5. A ResNet-50 network pretrained on ImageNet was used as a fixed feature extractor; all pretrained weights were frozen. The network was retained through the global average-pooling layer, yielding a 2048-dimensional feature vector for each slice. Slice-level feature vectors were averaged to obtain one patient-level representation for each region. The imaging models were therefore based on fixed deep features rather than end-to-end network training. The base random seed was 1314.

**Method S2.** Statistical comparison of model performance

All statistical comparisons and resampling procedures were conducted at the patient level. ROC-AUCs and their 95% confidence intervals were estimated using 10,000 outcome-stratified nonparametric bootstrap replicates. Within each replicate, patients with and without BM were resampled separately with replacement, while the paired predictions from all models were retained together for each patient.

Pairwise ROC-AUC differences between the DPPC model and each component model were evaluated using two-sided DeLong tests for correlated ROC curves. The DeLong covariance matrix and Z statistic were calculated using a custom paired_delong() function based on the fast DeLong algorithm. The Z statistic was calculated as the paired ROC-AUC difference divided by its DeLong standard error, and the corresponding two-sided P value was obtained from the standard-normal reference distribution.

The integrated discrimination improvement (IDI) was defined as the difference between the mean change in predicted probability among patients with BM and the corresponding mean change among patients without BM:

*IDI = mean(PDPPC − Preference | Y = 1) − mean(PDPPC − Preference | Y = 0)*

Positive IDI values indicate greater separation of predicted probabilities between patients with and without BM relative to the reference model. Percentile 95% confidence intervals were obtained from 10,000 patient-level outcome-stratified bootstrap replicates. Two-sided empirical P values were calculated as twice the smaller of the proportions of bootstrap IDI estimates on either side of zero, with a plus-one correction. The base random seed was 1314. Analyses were performed using Python 3.12.13, pandas 3.0.1, and NumPy 2.3.5. The corresponding functions were paired_delong() and stratified_bootstrap_idi().

# 2 Supplementary Algorithm

**Algorithm S1.** Patient-level nested model development, stacking, and meta-level cross-fitting

```
Input:
    Training cohort D
    Internal test cohort T
    Five patient-level outer folds
    Fixed ICC-eligible candidate feature sets

Base-model out-of-fold prediction:
    For each outer fold k = 1, ..., 5:
        Define Dtrain as patients in the other four folds
        Define Dholdout as patients in outer fold k

        Clinical pipeline:
            Fit the prespecified logistic-regression model using Dtrain
            Generate Clinical probabilities for Dholdout

        For region in {PG, PPAT}:
            Fit Z-score normalization using Dtrain
            Apply Benjamini-Hochberg-adjusted univariable screening in Dtrain
            Apply Pearson correlation filtering in Dtrain
            Select features by LASSO with inner cross-validation in Dtrain
            Tune SVM hyperparameters by inner cross-validation in Dtrain
            Fit Platt probability calibration using Dtrain only
            Generate calibrated probabilities for Dholdout

    Combine the OOF Clinical, PG, and PPAT probabilities for all patients in D

Meta-learner cross-fitting for training-cohort evaluation:
    For each meta-level fold k = 1, ..., 5:
        Fit the logistic-regression meta-learner using OOF component
            probabilities from patients in the other four folds
        Generate a DPPC probability for patients in fold k
    Pool the meta-level cross-fitted DPPC probabilities
    Calculate training-cohort performance

Final internal testing:
    Refit the three component pipelines using the entire training cohort D
    Fit the final meta-learner using all training-cohort OOF probabilities
    Generate Clinical, PG, and PPAT probabilities for T
    Apply the fixed meta-learner to obtain DPPC probabilities for T
    Evaluate T once without model or threshold refitting
```

# 3 Supplementary Tables

**Table S1.** Clinical characteristics according to BM status in the training and internal test cohorts

| **Variable** | **Training non-BM (n = 237)** | **Training BM (n = 87)** | **P value** | **Internal-test non-BM (n = 97)** | **Internal-test BM (n = 43)** | **P value** |
| --- | --- | --- | --- | --- | --- | --- |
| Age, years | 70.6 ± 8.4 | 70.2 ± 9.5 | 0.730 | 71.0 ± 8.3 | 70.2 ± 9.5 | 0.640 |
| BMI, kg/m² | 23.42 (21.23–25.46) | 22.76 (21.15–25.06) | 0.500 | 23.07 (20.76–25.34) | 23.38 (21.40–25.01) | 0.688 |
| tPSA, ng/mL | 16.02 (8.79–35.34) | 91.01 (24.98–135.00) | <0.001 | 16.82 (8.60–40.51) | 100.00 (31.55–191.13) | <0.001 |
| fPSA, ng/mL | 2.18 (1.15–5.01) | 12.48 (3.19–30.00) | <0.001 | 2.09 (1.40–5.90) | 20.60 (3.57–30.00) | <0.001 |
| f/tPSA | 0.14 (0.10–0.20) | 0.14 (0.10–0.29) | 0.409 | 0.13 (0.09–0.21) | 0.15 (0.10–0.22) | 0.427 |
| PSAD, ng/mL/cm³ | 0.40 (0.19–0.81) | 1.65 (0.60–3.53) | <0.001 | 0.48 (0.19–1.07) | 1.92 (0.96–3.65) | <0.001 |
| ALP, U/L | 64.00 (55.00–81.00) | 78.00 (65.00–120.00) | <0.001 | 64.00 (51.00–79.00) | 92.00 (65.50–146.00) | <0.001 |
| Prostate volume, cm³ | 40.56 (28.66–58.24) | 42.59 (30.81–56.87) | 0.847 | 39.45 (28.28–52.34) | 39.10 (31.43–54.62) | 0.412 |
| Normalized PPAT volume | 0.75 (0.58–1.07) | 1.16 (0.87–1.45) | <0.001 | 0.74 (0.59–0.94) | 1.08 (0.94–1.23) | <0.001 |
| Serum calcium, mmol/L | 2.32 (2.24–2.41) | 2.30 (2.20–2.44) | 0.434 | 2.33 (2.26–2.42) | 2.30 (2.19–2.41) | 0.222 |
| Serum phosphorus, mmol/L | 1.09 (0.98–1.21) | 1.09 (0.99–1.17) | 0.906 | 1.07 (0.97–1.22) | 1.07 (0.94–1.21) | 0.529 |
| **tPSA category, ng/mL** |  |  | **<0.001** |  |  | **<0.001** |
| <10 | 65 (27.4%) | 10 (11.5%) |  | 27 (27.8%) | 4 (9.3%) |  |
| 10–20 | 74 (31.2%) | 11 (12.6%) |  | 25 (25.8%) | 4 (9.3%) |  |
| >20 | 98 (41.4%) | 66 (75.9%) |  | 45 (46.4%) | 35 (81.4%) |  |
| **Clinical T stage** |  |  | **<0.001** |  |  | **<0.001** |
| 1 | 40 (16.9%) | 1 (1.1%) |  | 20 (20.6%) | 0 (0.0%) |  |
| 2 | 132 (55.7%) | 29 (33.3%) |  | 44 (45.4%) | 5 (11.6%) |  |
| 3 | 47 (19.8%) | 16 (18.4%) |  | 18 (18.6%) | 8 (18.6%) |  |
| 4 | 18 (7.6%) | 41 (47.1%) |  | 15 (15.5%) | 30 (69.8%) |  |
| **Clinical N stage** |  |  | **<0.001** |  |  | **<0.001** |
| 0 | 180 (75.9%) | 30 (34.5%) |  | 77 (79.4%) | 11 (25.6%) |  |
| 1 | 57 (24.1%) | 57 (65.5%) |  | 20 (20.6%) | 32 (74.4%) |  |
| **ISUP grade group** |  |  | **<0.001** |  |  | **<0.001** |
| 1 | 56 (23.6%) | 4 (4.6%) |  | 22 (22.7%) | 1 (2.3%) |  |
| 2 | 31 (13.1%) | 5 (5.7%) |  | 11 (11.3%) | 1 (2.3%) |  |
| 3 | 43 (18.1%) | 10 (11.5%) |  | 10 (10.3%) | 2 (4.7%) |  |
| 4 | 56 (23.6%) | 22 (25.3%) |  | 32 (33.0%) | 13 (30.2%) |  |
| 5 | 51 (21.5%) | 46 (52.9%) |  | 22 (22.7%) | 26 (60.5%) |  |
| **PI-RADS category** |  |  | **<0.001** |  |  | **<0.001** |
| 1 | 7 (3.0%) | 0 (0.0%) |  | 0 (0.0%) | 0 (0.0%) |  |
| 2 | 20 (8.4%) | 1 (1.1%) |  | 17 (17.5%) | 0 (0.0%) |  |
| 3 | 52 (21.9%) | 13 (14.9%) |  | 12 (12.4%) | 2 (4.7%) |  |
| 4 | 93 (39.2%) | 14 (16.1%) |  | 33 (34.0%) | 2 (4.7%) |  |
| 5 | 65 (27.4%) | 59 (67.8%) |  | 35 (36.1%) | 39 (90.7%) |  |
| **Diabetes** |  |  | **0.170** |  |  | **0.105** |
| No | 208 (87.8%) | 81 (93.1%) |  | 86 (88.7%) | 42 (97.7%) |  |
| Yes | 29 (12.2%) | 6 (6.9%) |  | 11 (11.3%) | 1 (2.3%) |  |
| **Hypertension** |  |  | **0.245** |  |  | **0.070** |
| No | 170 (71.7%) | 68 (78.2%) |  | 67 (69.1%) | 36 (83.7%) |  |
| Yes | 67 (28.3%) | 19 (21.8%) |  | 30 (30.9%) | 7 (16.3%) |  |
| **Coronary heart disease** |  |  | **0.937** |  |  | **0.776** |
| No | 209 (88.2%) | 77 (88.5%) |  | 85 (87.6%) | 39 (90.7%) |  |
| Yes | 28 (11.8%) | 10 (11.5%) |  | 12 (12.4%) | 4 (9.3%) |  |

*Age is reported as mean ± standard deviation; other continuous variables are reported as median (interquartile range). Categorical variables are reported as n (column %). There were no missing values for the variables shown. P values are descriptive and were not used for predictor selection. BM, bone metastasis; BMI, body mass index; tPSA, total prostate-specific antigen; fPSA, free prostate-specific antigen; PSAD, prostate-specific antigen density; ALP, alkaline phosphatase; PPAT, periprostatic adipose tissue; ISUP, International Society of Urological Pathology; PI-RADS, Prostate Imaging Reporting and Data System.*

**Table S2.** Pearson correlation matrix for the Clinical-model predictors in the training cohort

| **Predictor** | **log2(tPSA)** | **log2(ALP)** | **cT** | **cN** | **ISUP** |
| --- | --- | --- | --- | --- | --- |
| log2(tPSA) | 1.000 | 0.240 | 0.437 | 0.396 | 0.388 |
| log2(ALP) | 0.240 | 1.000 | 0.221 | 0.219 | 0.171 |
| cT | 0.437 | 0.221 | 1.000 | 0.513 | 0.479 |
| cN | 0.396 | 0.219 | 0.513 | 1.000 | 0.344 |
| ISUP | 0.388 | 0.171 | 0.479 | 0.344 | 1.000 |

*Correlations were calculated using the continuous or ordinal coding shown. cT, clinical T stage; cN, clinical N stage; ISUP, International Society of Urological Pathology grade group.*

**Table S3.** Spearman correlation matrix for the Clinical-model predictors in the training cohort

| **Predictor** | **tPSA** | **ALP** | **cT** | **cN** | **ISUP** |
| --- | --- | --- | --- | --- | --- |
| tPSA | 1.000 | 0.207 | 0.463 | 0.421 | 0.472 |
| ALP | 0.207 | 1.000 | 0.147 | 0.218 | 0.120 |
| cT | 0.463 | 0.147 | 1.000 | 0.502 | 0.486 |
| cN | 0.421 | 0.218 | 0.502 | 1.000 | 0.359 |
| ISUP | 0.472 | 0.120 | 0.486 | 0.359 | 1.000 |

*ALP, alkaline phosphatase; cT, clinical T stage; cN, clinical N stage; ISUP, International Society of Urological Pathology grade group.*

**Table S4.** Variance inflation factors for the Clinical-model design variables

| **Design variable** | **R²** | **VIF** |
| --- | --- | --- |
| cT_4 | 0.659 | 2.931 |
| cT_2 | 0.628 | 2.690 |
| ISUP_5 | 0.610 | 2.565 |
| cT_3 | 0.597 | 2.483 |
| ISUP_4 | 0.519 | 2.078 |
| tPSA_over_20 | 0.499 | 1.996 |
| ISUP_3 | 0.396 | 1.656 |
| tPSA_10_to_20 | 0.369 | 1.585 |
| cN | 0.324 | 1.479 |
| ISUP_2 | 0.321 | 1.473 |
| log2_ALP | 0.115 | 1.129 |

*The maximum VIF was 2.931. Indicator variables use the coding defined below Table S6. VIF, variance inflation factor.*

**Table S5.** Condition indices for the Clinical-model design matrix

| **Dimension** | **Eigenvalue** | **Condition index** |
| --- | --- | --- |
| 1 | 5.0708 | 1.000 |
| 2 | 1.7341 | 1.710 |
| 3 | 1.1345 | 2.114 |
| 4 | 1.0065 | 2.245 |
| 5 | 0.8726 | 2.411 |
| 6 | 0.7760 | 2.556 |
| 7 | 0.6700 | 2.751 |
| 8 | 0.3631 | 3.737 |
| 9 | 0.1664 | 5.519 |
| 10 | 0.1182 | 6.550 |
| 11 | 0.0812 | 7.902 |
| 12 | 0.0065 | 27.875 |

*The maximum condition index was 27.875.*

**Table S6.** Assessment of continuous-predictor functional form and complete Clinical-model equation

| **Predictor** | **Comparison** | **LR statistic** | **df** | **P value** |
| --- | --- | --- | --- | --- |
| log2(tPSA) | linear vs 4-df natural cubic spline | 11.197 | 3 | 0.011 |
| log2(ALP) | linear vs 4-df natural cubic spline | 1.198 | 3 | 0.753 |

*Likelihood-ratio tests compared a linear term with a four-degree-of-freedom natural cubic spline. LR, likelihood ratio; df, degrees of freedom.*

logit[P(BM=1)] =-11.1747245568 - 0.4268122229*tPSA_10_to_20 + 0.1968584042*tPSA_over_20 + 1.0866756439*log2_ALP + 2.0184936933*cT_2 + 1.9683833080*cT_3 + 3.3029061078*cT_4 + 0.7942394209*cN + 0.7133231132*ISUP_2 + 0.7514458439*ISUP_3 + 1.0000805426*ISUP_4 + 1.1020016604*ISUP_5
P(BM=1) = 1 / (1 + exp(-logit))
Reference categories: tPSA <10 ng/mL, cT1, and ISUP grade group 1. cT_k and ISUP_k are indicator variables for the stated category; cN is coded 0/1. tPSA_10_to_20 and tPSA_over_20 are indicator variables for 10-20 and >20 ng/mL, respectively. log2_ALP=log2(serum ALP).

**Table S7.** Overlap with previous institutional cohorts and data reuse

| **Previous study** | **Patients in current cohort** | **Proportion of current cohort** | **Reused material** | **Material generated for the present study** |
| --- | --- | --- | --- | --- |
| Normalized PPAT volume study (17) | 156 | 33.6% | Normalized PPAT volume and PPAT segmentation masks | PG segmentation masks; PG and PPAT ResNet-50 deep features; clinical component; DPPC stacking |
| PPAT radiomics study (36) | 151 | 32.5% | PPAT segmentation masks | PG segmentation masks; PG and PPAT ResNet-50 deep features; clinical component; DPPC stacking |
| Unique total overlap | 307 | 66.2% | As specified above | As specified above |

*The two previous cohorts did not overlap with each other. Previous handcrafted-radiomics signatures and fitted prediction models were not reused in the present analysis.*

**Table S8.** Sensitivity analysis of the DPPC model at the training-derived high-sensitivity threshold

| **Performance measure** | **Training cohort** | **Internal test cohort** |
| --- | --- | --- |
| Threshold | 0.109675 | 0.109675 |
| TP/TN/FP/FN | 79/174/63/8 | 41/70/27/2 |
| Sensitivity | 0.908 | 0.953 |
| Specificity | 0.734 | 0.722 |
| Accuracy | 0.781 | 0.793 |
| PPV | 0.556 | 0.603 |
| NPV | 0.956 | 0.972 |
| F1 score | 0.690 | 0.739 |
| Patients below threshold | 182 | 72 |
| BM rate below threshold | 4.4% | 2.8% |

*The threshold of 0.109675 was selected from training-cohort out-of-fold predictions as the highest threshold retaining sensitivity ≥90% and was applied unchanged to the internal test cohort. This analysis evaluates an alternative operating point and does not define a clinically established cutoff. PPV, positive predictive value; NPV, negative predictive value; BM, bone metastasis.*

Table S9. Main acquisition parameters of axial T2-weighted MRI

| **Parameter** | **Axial T2WI** |
| --- | --- |
| Sequence | Axial T2WI |
| TR (ms) | 4559 |
| TE (ms) | 110 |
| FOV (mm × mm) | 240 × 240 |
| Matrix | 320 × 224 |
| Slice thickness (mm) | 3 |
| Slice gap (mm) | 1 |
| NEX | 2 |
| Flip angle (°) | 111 |
| Bandwidth (kHz) | 41.67 |

Abbreviations: TR, repetition time; TE, echo time; FOV, field of view; NEX, number of excitations; T2WI, T2-weighted imaging.

# 4 Supplementary Figures

**Figure S1.** LASSO regression for PG region features selection.

(a) Cross-validation curve for selecting the optimal regularization parameter. (b) Coefficient profiles as the regularization parameter changed. (c) Selected PG deep features and their coefficients.

**Figure S2.** LASSO regression for PPAT region features selection.

(a) Cross-validation curve for selecting the optimal regularization parameter. (b) Coefficient profiles as the regularization parameter changed. (c) Selected PPAT deep features and their coefficients.
