# Supplementary material for: Dual-region deep learning model integrating prostate and peri-prostatic adipose tissue MRI features for bone metastasis prediction in prostate cancer
Source: Front Oncol. 2026 Sep 7;16:1946971. doi: 10.3389/fonc.2026.1946971 (PMC13590367; doi:10.3389/fonc.2026.1946971)
Supplement: Supplementary file 2 [file SupplementaryFile2.docx]

**Completed TRIPOD+AI Checklist**

**Dual-Region Deep Learning Model Integrating Prostate and Peri-prostatic Adipose Tissue MRI Features for Bone Metastasis Prediction in Prostate cancer**

Study type: Prediction-model development with internal evaluation

Location convention: section, table, figure, and supplementary identifiers are used because page numbers differ between the tracked and clean manuscript versions. 'Partial' and 'Not reported' entries are retained transparently and do not imply that unperformed analyses were conducted.

# Part A. TRIPOD+AI checklist for prediction model studies

| **Item** | **D/E** | **TRIPOD+AI checklist item** | **Location in revised submission and reporting note** |
| --- | --- | --- | --- |
| **Title** | | | |
| **1** | D;E | Identify the study as developing or evaluating the performance of a multivariable prediction model, the target population, and the outcome to be predicted | **Title page** **Status: Partial.** The model, population, and BM outcome are identified; development/internal evaluation is not stated explicitly in the title. |
| **Abstract** | | | |
| **2** | D;E | See TRIPOD+AI for Abstracts checklist | **Abstract; Part B of this checklist** **Status: Reported.** The abstract is mapped separately to the TRIPOD+AI for Abstracts checklist. |
| **Introduction** | | | |
| **3a** | D;E | Explain the healthcare context (including whether diagnostic or prognostic) and rationale for developing or evaluating the prediction model, including references to existing models | **Introduction, paragraphs 1-3** **Status: Reported.** Healthcare context, rationale, and previous BM prediction approaches are described. |
| **3b** | D;E | Describe the target population and the intended purpose of the prediction model in the context of the care pathway, including its intended users (eg, healthcare professionals, patients, public) | **Introduction, final paragraph; Discussion, clinical-use paragraph** **Status: Partial.** Target population and initial-staging purpose are described; intended users are not explicitly specified. |
| **3c** | D;E | Describe any known health inequalities between sociodemographic groups | **Not reported** **Status: Not reported.** No prespecified assessment of health inequalities across sociodemographic groups was performed. |
| **4** | D;E | Specify the study objectives, including whether the study describes the development or validation of a prediction model (or both) | **Introduction, final paragraph** **Status: Reported.** The stated objective is model development and internal evaluation for initial-staging BM risk assessment. |
| **Methods** | | | |
| **5a** | D;E | Describe the sources of data separately for the development and evaluation datasets (eg, randomised trial, cohort, routine care or registry data), the rationale for using these data, and representativeness of the data | **Methods - Patient Selection and Data Collection** **Status: Reported.** Retrospective single-centre cohort data were randomly divided into training and internal test cohorts. |
| **5b** | D;E | Specify the dates of the collected participant data, including start and end of participant accrual; and, if applicable, end of follow-up | **Methods - Patient Selection and Data Collection** **Status: Reported.** Participant accrual dates (January 2014-December 2024) and analysis timing are reported. |
| **6a** | D;E | Specify key elements of the study setting (eg, primary care, secondary care, general population) including the number and location of centres | **Title-page affiliation; Methods - Patient Selection and Data Collection** **Status: Reported.** Single-centre tertiary urology setting and institutional location are identified. |
| **6b** | D;E | Describe the eligibility criteria for study participants | **Methods - Patient Selection and Data Collection; Figure 1** **Status: Reported.** Eligibility and exclusion criteria are described. |
| **6c** | D;E | Give details of any treatments received, and how they were handled during model development or evaluation, if relevant | **Methods - Patient Selection and Data Collection; Reference Standard and Study Chronology** **Status: Reported.** Patients were evaluated at initial staging before treatment; previous neoadjuvant therapy was an exclusion criterion. |
| **7** | D;E | Describe any data pre-processing and quality checking, including whether this was similar across relevant sociodemographic groups | **Methods - MRI Acquisition; Image Preprocessing and Deep Learning Feature Extraction; Feature Selection and Model Construction; Supplementary Method S1** **Status: Partial.** Image resizing, intensity normalisation, feature preprocessing, and fold-contained procedures are reported; sociodemographic-group-specific preprocessing checks were not performed. |
| **8a** | D;E | Clearly define the outcome that is being predicted and the time horizon, including how and when assessed, the rationale for choosing this outcome, and whether the method of outcome assessment is consistent across sociodemographic groups | **Methods - Reference Standard and Study Chronology** **Status: Reported.** BM at initial staging, SPECT/CT assessment, chronology, equivocal-case handling, and ancillary confirmation are described. |
| **8b** | D;E | If outcome assessment requires subjective interpretation, describe the qualifications and demographic characteristics of the outcome assessors | **Methods - Reference Standard and Study Chronology** **Status: Partial.** Reader number and experience are reported; reader demographic characteristics are not reported. |
| **8c** | D;E | Report any actions to blind assessment of the outcome to be predicted | **Methods - Reference Standard and Study Chronology** **Status: Reported.** Outcome readers were blinded to MRI findings, clinical information, model outputs, and each other's assessments. |
| **9a** | D | Describe the choice of initial predictors (eg, literature, previous models, all available predictors) and any pre-selection of predictors before model building | **Methods - Clinical Model Construction; Feature Selection and Model Construction** **Status: Reported.** Clinical predictors were prespecified by clinical relevance; imaging candidates underwent reproducibility and fold-contained selection. |
| **9b** | D;E | Clearly define all predictors, including how and when they were measured (and any actions to blind assessment of predictors for the outcome and other predictors) | **Methods - Clinical Model Construction; MRI Acquisition and ROI Segmentation; Supplementary Tables S1-S6** **Status: Partial.** Predictor definitions, transformations, and coding are reported; timing of every laboratory measurement is not separately specified. |
| **9c** | D;E | If predictor measurement requires subjective interpretation, describe the qualifications and demographic characteristics of the predictor assessors | **Methods - MRI Acquisition and ROI Segmentation; Volumetric Quantification and Reliability** **Status: Partial.** Radiologist experience and segmentation workflow are reported; assessor demographic characteristics are not reported. |
| **10** | D;E | Explain how the study size was arrived at (separately for development and evaluation), and justify that the study size was sufficient to answer the research question. Include details of any sample size calculation | **Methods - Patient Selection and Data Collection; Results - Patient Characteristics; Figure 1** **Status: Partial.** All eligible patients during the study period were included and cohort/event counts are reported; no a priori sample-size calculation was performed. |
| **11** | D;E | Describe how missing data were handled. Provide reasons for omitting any data | **Supplementary Table S1 footnote** **Status: Reported.** No missing values were present for the displayed analysis variables; no imputation procedure was required. |
| **12a** | D | Describe how the data were used (eg, for development and evaluation of model performance) in the analysis, including whether the data were partitioned, considering any sample size requirements | **Methods - Patient Selection and Data Collection; Feature Selection and Model Construction; Probability Stacking** **Status: Reported.** The 7:3 patient-level split, five-fold outer procedure, and meta-level cross-fitting are described. |
| **12b** | D | Depending on the type of model, describe how predictors were handled in the analyses (functional form, rescaling, transformation, or any standardisation) | **Methods - Clinical Model Construction; Feature Selection and Model Construction; Supplementary Tables S2-S6** **Status: Reported.** Categorisation, transformation, Z-score normalisation, and functional-form assessment are described. |
| **12c** | D | Specify the type of model, rationale†, all model building steps, including any hyperparameter tuning, and method for internal validation | **Methods - Clinical Model Construction; Feature Selection and Model Construction; Probability Stacking; Figure 2; Algorithm S1** **Status: Reported.** Logistic regression, SVM development, hyperparameter tuning, Platt calibration, stacking, and internal validation are described. |
| **12d** | D;E | Describe if and how any heterogeneity in estimates of model parameter values and model performance was handled and quantified across clusters (eg, hospitals, countries). See TRIPOD-Cluster for additional considerations‡ | **Not applicable** **Status: N/A.** The analysis used one centre and did not model clustering across hospitals or countries. |
| **12e** | D;E | Specify all measures and plots used (and their rationale) to evaluate model performance (eg, discrimination, calibration, clinical utility) and, if relevant, to compare multiple models | **Methods - Model Evaluation and Interpretability; Statistical Analysis; Supplementary Method S2** **Status: Reported.** Discrimination, calibration, classification, clinical utility, DeLong, and IDI analyses are specified. |
| **12f** | E | Describe any model updating (eg, recalibration) arising from the model evaluation, either overall or for particular sociodemographic groups or settings | **Not applicable** **Status: N/A.** No model updating or recalibration was performed after evaluation. |
| **12g** | E | For model evaluation, describe how the model predictions were calculated (eg, formula, code, object, application programming interface) | **Methods - Probability Stacking; Algorithm S1; Supplementary Table S6** **Status: Partial.** Prediction generation is described and the Clinical-model equation is supplied; complete deployable PG, PPAT, and DPPC model objects are not embedded in the manuscript. |
| **13** | D;E | If class imbalance methods were used, state why and how this was done, and any subsequent methods to recalibrate the model or the model predictions | **Methods - Feature Selection and Model Construction** **Status: Reported.** SVM class_weight='balanced' and subsequent Platt probability calibration are reported. |
| **14** | D;E | Describe any approaches that were used to address model fairness and their rationale | **Not reported** **Status: Not reported.** No formal model-fairness analysis across sociodemographic groups was performed. |
| **15** | D | Specify the output of the prediction model (eg, probabilities, classification). Provide details and rationale for any classification and how the thresholds were identified | **Methods - Model Evaluation; Results - Model Calibration and Clinical Usefulness; Supplementary Table S8** **Status: Reported.** The primary output is probability; the descriptive 0.5 threshold and training-derived high-sensitivity sensitivity analysis are described. |
| **16** | D;E | Identify any differences between the development and evaluation data in healthcare setting, eligibility criteria, outcome, and predictors | **Table 1; Results - Patient Characteristics and Recruitment** **Status: Reported.** Overall training/internal-test differences are reported, including differences in cT and PI-RADS distributions. |
| **17** | D;E | Name the institutional research board or ethics committee that approved the study and describe the participant informed consent or the ethics committee waiver of informed consent | **Methods - Patient Selection and Data Collection** **Status: Reported.** Institutional review board approval number and waiver of informed consent are reported. |
| **Open science** | | | |
| **18a** | D;E | Give the source of funding and the role of the funders for the present study | **Funding** **Status: Partial.** Funding sources and grant numbers are reported; the role of the funders is not explicitly stated. |
| **18b** | D;E | Declare any conflicts of interest and financial disclosures for all authors | **Conflict of Interest** **Status: Reported.** The authors declare no relevant commercial or financial relationships. |
| **18c** | D;E | Indicate where the study protocol can be accessed or state that a protocol was not prepared | **Not reported** **Status: Not reported.** A separate study protocol was not prepared, but this is not stated in the manuscript. |
| **18d** | D;E | Provide registration information for the study, including register name and registration number, or state that the study was not registered | **Not reported** **Status: Not reported.** The retrospective prediction-model study was not registered, but this is not stated in the manuscript. |
| **18e** | D;E | Provide details of the availability of the study data | **Not reported in the manuscript source file** **Status: Not reported.** A data-availability statement remains to be completed in the Frontiers submission system. |
| **18f** | D;E | Provide details of the availability of the analytical code§ | **Not reported in the manuscript source file** **Status: Not reported.** No permanent code repository or access link is currently provided. |
| **Patient and public involvement** | | | |
| **19** | D;E | Provide details of any patient and public involvement during the design, conduct, reporting, interpretation, or dissemination of the study or state no involvement | **Not reported** **Status: Not reported.** No patient or public involvement was undertaken, but this is not stated in the manuscript. |
| **Result** | | | |
| **20a** | D;E | Describe the flow of participants through the study, including the number of participants with and without the outcome and, if applicable, a summary of the follow-up time. A diagram may be helpful | **Results - Patient Characteristics and Recruitment; Figure 1** **Status: Reported.** Participant flow and numbers with and without BM are reported. |
| **20b** | D;E | Report the characteristics overall and, where applicable, for each data source or setting, including the key dates, key predictors (including demographics), treatments received, sample size, number of outcome events, follow-up time, and amount of missing data. A table may be helpful. Report any differences across key demographic groups | **Table 1; Supplementary Table S1; Results - Patient Characteristics and Recruitment** **Status: Partial.** Cohort characteristics, event counts, and BM-stratified characteristics are reported; patient-level scanner/calendar-period distributions are unavailable. |
| **20c** | E | For model evaluation, show a comparison with the development data of the distribution of important predictors (demographics, predictors, and outcome) | **Table 1; Results - Patient Characteristics and Recruitment** **Status: Reported.** Training and internal test cohorts are compared directly. |
| **21** | D;E | Specify the number of participants and outcome events in each analysis (eg, for model development, hyperparameter tuning, model evaluation) | **Methods - Patient Selection; Results - Patient Characteristics; Figure 1** **Status: Partial.** Training and internal-test participant/event counts are reported; fold-specific event counts are not tabulated. |
| **22** | D | Provide details of the full prediction model (eg, formula, code, object, application programming interface) to allow predictions in new individuals and to enable third party evaluation and implementation, including any restrictions to access or reuse (eg, freely available, proprietary)¶ | **Table 2; Supplementary Table S6** **Status: Partial.** The complete Clinical-model equation is supplied; deployable PG, PPAT, and DPPC model objects are not supplied in the submitted files. |
| **23a** | D;E | Report model performance estimates with confidence intervals, including for any key subgroups (eg, sociodemographic). Consider plots to aid presentation | **Table 3; Figures 3-5; Results - Model Construction and Performance; Model Calibration and Clinical Usefulness** **Status: Partial.** Performance estimates and bootstrap confidence intervals are reported; prespecified sociodemographic subgroup performance was not evaluated. |
| **23b** | D;E | If examined, report results of any heterogeneity in model performance across clusters. See TRIPOD-Cluster for additional details‡ | **Not applicable** **Status: N/A.** Cluster-level heterogeneity was not examined in this single-centre dataset. |
| **24** | E | Report the results from any model updating, including the updated model and subsequent performance | **Not applicable** **Status: N/A.** No model updating was performed. |
| **Discussion** | | | |
| **25** | D;E | Give an overall interpretation of the main results, including issues of fairness in the context of the objectives and previous studies | **Discussion, paragraphs 1-7** **Status: Partial.** Results are interpreted in relation to study objectives and previous studies; fairness was not evaluated. |
| **26** | D;E | Discuss any limitations of the study (such as a non-representative sample, sample size, overfitting, missing data) and their effects on any biases, statistical uncertainty, and generalisability | **Discussion - Limitations** **Status: Reported.** Single-centre design, internal random split, event count, cohort differences, scanner/protocol heterogeneity, manual segmentation, and reference-standard limitations are discussed. |
| **27a** | D | Describe how poor quality or unavailable input data (eg, predictor values) should be assessed and handled when implementing the prediction model | **Not reported** **Status: Not reported.** Procedures for handling unavailable or poor-quality inputs during implementation are not specified. |
| **27b** | D | Specify whether users will be required to interact in the handling of the input data or use of the model, and what level of expertise is required of users | **Not reported** **Status: Not reported.** User interaction and expertise requirements for future implementation are not specified. |
| **27c** | D;E | Discuss any next steps for future research, with a specific view to applicability and generalisability of the model | **Discussion - clinical-use paragraph and Limitations; Conclusion** **Status: Reported.** External multicentre validation, calibration assessment, and further clinical-utility evaluation are identified as next steps. |

# Part B. TRIPOD+AI for Abstracts checklist

| **Item** | **TRIPOD+AI checklist item** | **Location in revised submission and reporting note** |
| --- | --- | --- |
| **Title** | | |
| **1** | Identify the study as developing or evaluating the performance of a multivariable prediction model, the target population, and the outcome to be predicted | **Title page** **Status: Partial.** The model, population, and outcome are identified, but development/internal evaluation is not explicit. |
| **Background** | | |
| **2** | Provide a brief explanation of the healthcare context and rationale for developing or evaluating the performance of all models | **Abstract - Rationale and Objectives** **Status: Reported.** Clinical context and rationale are stated. |
| **Objectives** | | |
| **3** | Specify the study objectives, including whether the study describes model development, evaluation, or both | **Abstract - Rationale and Objectives** **Status: Reported.** Development and internal evaluation of a multimodal BM risk model are described. |
| **Methods** | | |
| **4** | Describe the sources of data | **Abstract - Methods** **Status: Reported.** Retrospective cohort source and 7:3 training/internal-test split are stated. |
| **5** | Describe the eligibility criteria and setting where the data were collected | **Abstract - Methods** **Status: Partial.** Participant count and retrospective design are stated; detailed eligibility and setting appear in the main Methods. |
| **6** | Specify the outcome to be predicted by the model, including time horizon of predictions in case of prognostic models | **Abstract - Rationale and Objectives/Methods** **Status: Reported.** BM risk assessment at initial staging is the target outcome/context. |
| **7** | Specify the type of model, a summary of the model-building steps, and the method for internal validation† | **Abstract - Methods** **Status: Reported.** ResNet-50 fixed deep features, component models, out-of-fold probabilities, and logistic-regression stacking are summarised. |
| **8** | Specify the measures used to assess model performance (eg, discrimination, calibration, clinical utility) | **Abstract - Methods** **Status: Reported.** ROC-AUC, AP, calibration, decision-curve, and SHAP analyses are listed. |
| **Results** | | |
| **9** | Report the number of participants and outcome events | **Abstract - Methods/Results** **Status: Partial.** Total participants are reported; cohort-specific BM event counts are not included in the abstract. |
| **10** | Summarise the predictors in the final model† | **Abstract - Methods** **Status: Reported.** PG and PPAT deep features and clinical predictors are summarised. |
| **11** | Report model performance estimates (with confidence intervals) | **Abstract - Results** **Status: Reported.** ROC-AUC estimates with 95% confidence intervals and threshold performance are reported. |
| **Discussion** | | |
| **12** | Give an overall interpretation of the main results | **Abstract - Conclusion** **Status: Reported.** The conclusion interprets performance and notes the need for external validation and calibration assessment. |
| **Registration** | | |
| **13** | Give the registration number and name of the registry or repository | **Not applicable** **Status: N/A.** The retrospective study was not registered. |

Checklist source: Collins GS, Moons KGM, Dhiman P, et al. TRIPOD+AI statement: updated guidance for reporting clinical prediction models that use regression or machine learning methods. BMJ. 2024;385:e078378. doi:10.1136/bmj-2023-078378.
